# Mental health status of children with disorders of sexual development and their correlates

**DOI:** 10.3389/fpubh.2022.756382

**Published:** 2022-07-27

**Authors:** Jingjing Cai, Guochun Zhu, Hongjuan Tian, Jinna Yuan, Huihui Gao, Liying Sun, Guanping Dong, Wei Ru, Dehua Wu, Daxing Tang, Weijia Gao, Junfen Fu, Rongwang Yang

**Affiliations:** ^1^Department of Child Psychology, Children's Hospital, National Clinical Research Center for Child Health, Zhejiang University School of Medicine, Hangzhou, China; ^2^Department of Urology, Children's Hospital, National Clinical Research Center for Child Health, Zhejiang University School of Medicine, Hangzhou, China; ^3^Department of Endocrinology, Children's Hospital, National Clinical Research Center for Child Health, Zhejiang University School of Medicine, Hangzhou, China; ^4^Department of Pediatrics and Adolescent Gynecology, Children's Hospital, National Clinical Research Center for Child Health, Zhejiang University School of Medicine, Hangzhou, China

**Keywords:** disorders of sexual development, anxiety, parental rearing practices, family environment, mental health

## Abstract

Disorders of sexual development (DSD) refer to the congenital abnormalities of chromosomes, gonads, or gender anatomy. Children with DSD usually experience more stress. The present study aims to evaluate the mental health status of children with DSD, and to explore the potential relevant factors. We included 30 children with DSD and 30 age- and gender-matched children without DSD as the control group. All the children and their parents completed the scales of the Hamilton Anxiety Scale (HAMA). Children over 8 years old (*n* = 22) completed the Screen Scale for Child Anxiety Related Emotional Disorders (SCARED), the Depression Self-rating Scale for Children (DSRSC), and the Egna Minnen av Barndoms Uppfostran-own memories of parental rearing practices in childhood. DSD children had significantly higher somatic anxiety, mental anxiety, and total anxiety scores than the control group (*p* < 0.001). The scores of the SCARED, anxiety, and depression subscales of DSD children were higher than those of control children (*p* < 0.05 and *p* < 0.001, respectively). The correlation analysis showed that the score of generalized anxiety was positively related to age and entertainment. The regression analysis showed that age was a major factor that affected generalized anxiety in DSD children, and neuroticism was a major factor of anxiety disorder and separation anxiety in DSD children. Children with DSD have obvious anxiety problems, which are associated with family environmental factors (entertainment, success, and conflicts) and age. It is important to focus emphasis on emotional stability in children with DSD for detecting anxiety-related emotional disorders early.

## Introduction

Disorders of sexual development (DSD) are a group of disorders classified by congenital abnormalities of chromosomes, gonads, or gender anatomy, mostly seen in newborns and adolescents (1–3). DSDs have a wide spectrum, and patients with different pathophysiological changes present with different clinical manifestations (1–3). Newborns with DSDs normally present with genital abnormalities, while adolescents with abnormal sexual development present during youth development (4). DSDs are divided into three categories: 46, XX (mainly related to SRY gene translocation, androgen excess), 46, XY (mainly related to gao differentiation development, androgen synthesis, and utilization disorders), and sex chromosome DSD (mainly related to sex chromosomal karyotype abnormalities) (1–5).

Children and adolescents with DSDs are at high risk of emotional problems. The relevant factors affecting emotional problems mainly focus on three aspects: genetic factors, psychological factors, and social environmental factors.

Ediati A et al. (6) compared 118 Indonesian DSD patients aged 6–41 years (60 children, 24 adolescents, and 34 adults) with 118 healthy control subjects matched for age, gender, and residential settings. The results of the Child Behavioral Checklist (CBCL) showed that parents of DSD children had significantly more emotional and behavioral problems than those of normal children. The results of the Adult Self-Report (ASR) indicated that adults with DSD had significantly more internalizing problems, particularly anxiety and depression, than the control group. The research suggested that Indonesian patients with DSD who had not been treated for most of their lives suffered from more emotional and behavioral problems than matched controls. Kleinemeier E et al. (7) studied 60 DSD teenagers aged 13–16 years old using standardized instruments and scales covering health-related quality of life, mental health, physical image, and other issues related to sexual behavior and response to DSDs. Compared to adolescents who entered puberty spontaneously, those who needed hormonal treatment to induce puberty have been reported to be affected in nearly all outcomes. It has been reported that girls with DSDs have less sexual activity than female controls.

It is stated that children with these disorders have high levels of stress. The timing of diagnosis seems to specifically contribute to the increased risk of developing negative feelings, intense reactions, or acute psychological conditions (8). These children may face confusion about how to accept and adapt to the opposite sex lifestyle, especially self-identity regarding body image. They would worry or even fear using the toilet in public. Children might have little idea how to live with peers of the same age with a new gender since they are afraid of too much attention from others. The individuation process of adolescence and its close relationship with peer identification increase the sensibility toward the pressure of normality or conformity (5).

Children may find it difficult to explain DSDs to their classmates and teachers, which might hinder the development of their social behavior (5). Children are in the acute stress response stage and tend to become sensitive and anxious. If there is no reasonable way to deal with their feelings, they may gradually develop serious emotional problems. Appropriate interventions would be necessary to prevent worse outcomes of social functioning impairment for these children. Sexual orientation is still a sensitive topic in Chinese families. Traditional Chinese families often found it hard to accept their children's sudden gender change, especially in the son-preference families. In addition, DSD may bring many challenges in terms of changes in family parenting style, education policies, and parent–child relationship, leading to difficulties in psycho-social aspects of children and their families beyond the disorder itself. In China, there are few studies on this topic. Therefore, the present study aims to evaluate the mental health status of children with DSD, and to explore the potential relevant factors.

## Methods

This is a cross-sectional and case-control investigation. The recruited participants were children and adolescents aged 3–17 years diagnosed with DSD in the joint clinic from June 2019 to June 2021. The diagnostic criteria for DSD were the consensus of Chinese experts (2, 3). The criteria included that the shape of external genitalia is blurred, and two sets of reproductive systems are revealed by the B-scan ultrasonography or endoscopic exploration, or the chromosomes are not consistent with the social sex. The common feature of all DSDs was that both male and female choices were available for sex determination. The participants and their parents were interviewed with the Hamilton Anxiety (HAMA) scale. Semi-structural interviews and observations were based on the Hamilton scale to evaluate children under the age of 8 years covering the symptoms of worrying, nervousness, phobia, sleep problems, cognitive functions, depressive feelings, physical anxiety, sensory system, cardiovascular system, respiratory and digestive system, reproduction and urinary symptoms, autonomic nervous system, and relatively comprehensive evaluation of general performance, and physiological performance during the conversation (9). Children over 8 years old completed the Screen for Child Anxiety Related Emotional Disorders (SCARED), the Depression Self-rating Scale for Children (DSRSC), and the Egna Minnen av Barndoms Uppfostran-own memories of parental rearing practices in childhood (EMBU). Typically developing children and adolescents with age and gender matching were recruited as a control group. They were excluded if they had any known serious medical disorders, such as depression, anxiety disorder, and other mental disorders; and their parents were without serious mental health disorders.

### Measures

We collected children's gender, age, physical development, and health history, the age and educational level of the parents; the pregnancy history of the mother, and the economic status of the family using questionnaires.

The HAMA, SCARED, and DSRSC were used to evaluate the mental health status of participants. HAMA was scored independently by two trained assessors through conversation and observation. HAMA divides anxiety factors into somatic and psychological factors (9). The total HAMA score can better reflect the severity of the anxiety symptoms, with a reliability coefficient of 0.93 and validity coefficient of 0.36 (10). Children with a total score higher than 7 are defined as having anxiety; those with a total score <7 are defined as having no anxiety symptoms.

The SCARED scale was compiled by Birmaher with 41 entries. Five of these factors are parallel with anxiety disorders in the DSM-IV. Based on the children's self-assessment, the Chinese urban permanent model was revised in 2002 (11).

The DSRSC was compiled by Birleson, with 18 entries. This scale can be completed by children, and a score higher than 15 indicated that the participating children were in a depressed state (12).

The home environment scale-Chinese version (FES-CV) scale consists of 10 component tables that evaluate 10 different family social and environmental characteristics. The scale contains 90 true-false questions and has good validity, parametric reliability, and high internal consistency confidence in the affinity, ambivalence, knowledge, and organization. The individual reliability coefficients of the scale were above 0.70. The scale is available for children who have a degree above primary education (13, 14).

The EMBU was to evaluate parenting methods through recollection. EMBU has 81 entries and 2 additional entries, involving 15 parenting behaviors and four main factors: rejection, emotional warmth, excessive protection, and preference (15).

### Statistical analysis

Data were analyzed using SPSS 22.0. The sample size of this project was calculated by the formula of *N* = *Z*^2^ × (*p* × (1 – *p*))/*E*^2^. Thirty was the minimum sample size for quantitative research. The normality of data was tested by Kolmogorov–Smirnov test in SPSS analysis, resulting in non-normality (*p* < 0.05), except for the age of 8 years or older and total scores of SCARED and DSRSC (*p* > 0.05). Variables with normal distribution were tested with *T*-test, and variables with non-normal distribution were tested with non-parametric tests. The Pearson correlation analysis was used to analyze the relationships between mental health status, age, and some of the family factors in DSD children. Multiple linear regression analysis was performed to explore the related factors associated with DSDs in children's emotional states. All tests were two tailed and a *p*-value of <0.05 was considered to be statistically significant.

### Ethics approval and informed consent

This study was approved by the Ethics Committee of the Children's Hospital affiliated to Zhejiang University of Medicine. All parents or guardians of the included children provided written informed consent for screening and clinical treatment.

## Results

### Sample characteristics of the children

Thirty children with DSDs were recruited in the study. The control group included 30 typical developing children from a kindergarten, a primary school, and a middle school in Hangzhou. The average age of children in the DSD and control groups was 10.36 ± 3.50 years and 10.37 ± 3.47 years, respectively. Both groups consisted of 2 boys (6.67%) and 28 girls (93.33%). The average age of children older than 8 years old in the two groups (22 children with 21 women and a male) was 12.08 ± 2.22 years and 12.09 ± 2.16 years, respectively.

In the DSD group, nineteen of them had confirmed SRY mutations, 5 had SRD5A2 mutations, 2 had CYP17A1 mutations, and 11 had not completed relevant genetic tests. One female chromosome was (46, XX, SRY gene: negative) and one female chromosome was (46, XY [40] / 45, X (13)). Only 2 of the remaining 28 individuals (46, XY) were male.

The total HAMA score for all children in the DSD group indicated their level of anxiety was 8.10 ± 5.57, while for the control group it was 1.50 ± 1.55. Fifteen children (50%) in the DSDs group had a total HAMA score higher than 6, which suggested possible anxiety symptoms. However, no one in the control group reached a total HAMA score of 7. The total scores of SCARED and DSRSC for DSD group were 20.64 ± 12.76 and 9.32 ± 6.27, respectively. For the control group, the total scores of SCARED and DSRSC were 6.95 ± 3.63 and 2.91 ± 2.16, respectively.

### Comparison of depression and anxiety disorder between DSD children and control children

Children in the DSD group had significantly higher somatic anxiety, psychic anxiety, and total anxiety scores than the control group (*p* < 0.001). Children with DSD had higher scores for subscales of somatization/phobia, generalized anxiety, separation anxiety, social phobia, school phobia, and higher total scores of anxiety and depression than the control group (*p* < 0.05 and *p* < 0.001, respectively). In addition, DSD children had more severe psychological anxiety than somatic anxiety. The children with DSDs had more pronounced anxiety and depression than children without DSDs (Table 1).

**Table 1:** Comparison of the he results of HAMA, SCARED, and DSRSC between DSDs children and controls.

**Characteristic**	**Children with DSD**	**The control group**	**Statistic**	***P*** **-value**
All children	*n* = 30	*n* = 30		
Age	10.36 ± 3.50	10.37 ± 3.47	*Z = −0.158*	*0.875*
Gender				
Boy	2	2		
Girl	28	28		
Total score of HAMA	8.10 ± 5.57	1.50 ± 1.55	*Z = −4.645*	* < 0.001*
Somatic anxiety	2.17 ± 2.34	0.57 ± 0.63	*Z = −3.327*	*0.001*
Psychic anxiety	5.97 ± 3.96	0.87 ± 1.04	*Z = −4.631*	* < 0.001*
Children over 8 years old	*n* = 22	*n* = 22		
Age	12.08 ± 2.22	12.09 ± 2.16	*t* = −0.143	0.887
SCARED	20.64 ± 12.76	6.95 ± 3.63	*t* = 4.589	<0.001
Somatization/phobia	4.95 ± 3.51	1.68 ± 1.46	*Z = 3.089*	*0.002*
Generalized anxiety	5.45 ± 4.84	1.14 ± 0.77	*Z = −3.424*	*0.001*
Separative anxiety	4.45 ± 3.39	1.41 ± 1.40	*Z = −3.012*	*0.003*
Social phobia	4.41 ± 3.03	2.32 ± 2.40	*Z = −2.080*	*0.038*
School phobia	1.32 ± 1.25	0.41 ± 0.67	*Z = −2.632*	*0.008*
Total score of DSRSC	9.32 ± 6.27	2.91 ± 2.16	*t* = 4.466	<0.001

### Relationship between anxiety, depression, age, and the FES-CV in children with DSDs

In children with DSDs, age and family entertainment in FES-CV were positively correlated with generalized anxiety score in SCARED (*r* = 0.44, *p* < 0.05 and *r* = 0.34, *p* < 0.05, respectively). Family success was negatively correlated with social phobia score (*r* = −0.37, *P* < 0.05). Family contradiction was positively associated with both school phobia score in SCARED, and total DSRSC score (*r* = 0.37, *p* < 0.05 and *r* = 0.36, *p* < 0.05, respectively). No significant correlations were found among the remaining variables. Those results suggested that the DSD children's emotions were associated with some family environmental factors (entertainment, success, contradiction) and age (*P* < 0.05) (Table 2).

**Table 2:** Correlation analysis of anxiety and depression, age and the FES-CV in children with DSD.

	**Somatization** **/phobia (*****n** **=*** **22)**	**Generalized anxiety (*****n** **=*** **22)**	**Separative anxiety (*****n** **=*** **22)**	**Social phobia (*****n** **=*** **22)**	**School phobia (*****n** **=*** **22)**	**Total score of SCARED (*****n** **=*** **22)**	**Total score of DSRSC (*****n** **=*** **22)**	**Total score of HAMA (*****n** **=*** **30)**
Age	–0.2 (0.46)	0.44 (0.02)*	–0.30 (0.08)	0.17 (0.22)	0.20 (0.19)	0.13 (0.28)	0.26 (0.12)	0.36 (0.05)
Intimacy	–0.09 (0.34)	0.08 (0.36)	–0.08 (0.37)	0.04 (0.44)	–0.09 (0.35)	–0.01 (0.48)	–0.06 (0.89)	–0.23 (0.22)
Emotional Expression	0.06 (0.40)	0.03 (0.45)	0.08 (0.36)	0.08 (0.36)	–0.07 (0.37)	0.07 (0.39)	0.07 (0.38)	0.09 (0.64)
Conflicts	–0.07 (0.38)	0.19 (0.20)	0.15 (0.26)	–0.12 (0.30)	0.37 (0.05)*	0.10 (0.34)	0.36 (0.05)*	0.06 (0.76)
Independence	–0.16 (0.24)	–0.05 (0.40)	0.01 (0.48)	–0.17 (0.22)	0.00 (0.49)	–0.10 (0.38)	0.08 (0.36)	–0.36 (0.05)
Success	0.01 (0.47)	–0.32 (0.70)	–0.00 (0.49)	–0.37 (0.04)*	–0.18 (0.22)	–0.23 (0.15)	–0.11 (0.32)	0.16 (0.40)
Intellectual	–0.19 (0.20)	0.04 (0.44)	–0.14 (0.27)	–0.19 (0.20)	0.04 (0.43)	–0.12 (0.29)	–0.18 (0.21)	0.04 (0.84)
Entertaining	0.23 (0.15)	0.37 (0.05)*	0.13 (0.29)	0.33 (0.07)	0.07 (0.37)	0.32 (0.07)	0.26 (0.12)	0.18 (0.35)
Moral and Religious views	–0.10 (0.33)	–0.13 (0.28)	0.23 (0.15)	–0.28 (0.11)	0.12 (0.30)	–0.08 (0.36)	0.09 (0.35)	–0.00 (0.99)
Organizational	–0.22 (0.16)	0.02 (0.46)	0.01 (0.49)	0.06 (0.39)	0.14 (0.27)	–0.02 (0.46)	–0.11 (0.32)	–0.19 (0.32)
Controlling	–0.18 (0.21)	–0.21 (0.18)	–0.04 (0.43)	–0.32 (0.07)	–0.03 (0.45)	–0.22 (0.16)	–0.10 (0.32)	0.36 (0.05)

Data were presented as r (p). ^*^P < 0.05. The bold values meant that the difference between the two factors was significant.

### Regression analysis of total anxiety and depression in DSD children

The total scores of depression and anxiety in DSD children were divided into factor variables. Age and family environment (entertainment, success, and contradiction) were selected as independent variables. Linear regression analysis showed that age was a major factor that affected generalized anxiety in DSD children (Table 3). The effect of age on the generalized anxiety score of SCARED was found to be statistically significant (*B* = 0.963, *p* < 0.05). For total scores of HAMA, SCARED,DSRSC, and subscale score of SCARED, no significant results were found for any other variables (*p* > 0.05).

**Table 3:** Regression analysis of factors related to anxiety and depression in DSDs children.

	**Variable**	***B***	**Standard error**	**Beta**	***T***	***P*-value**
**Generalized anxiety**	**Constant**	−6.186	5.358		−1.155	0.262
	**Age**	0.963	0.436	0.443	2.207	0.039
**Total score of the HAMA**	**Constant**	−0.517	4.589		−0.113	0.911
**Separative anxiety**	**Constant**	−1.851	2.851		−0.649	0.524

## Discussion

### Main findings

Children with DSD whose total HAMA score was higher than 7 points might have anxiety problems. The problem of psychological anxiety in the children with DSDs was more serious than somatic anxiety in our findings, suggesting that children with DSDs were more likely to suffer from anxiety and depression than children without DSDs. Children with DSDs had obvious anxiety-related emotional problems, and it was associated with family environment factors (entertainment, success, and contradiction) and age. Therefore, age in children with DSDs can help with the early detection of emotional disorders and anxiety-related emotional disorders.

### Importance

The findings of this study highlighted the fact that children with DSDs have relatively worse mental health status in terms of emotional conditions, such as anxiety and depressive symptoms. It confirmed the high prevalence of anxiety and depression in children with DSDs, which has been previously reported in various populations in many previous empirical studies (16). Our findings that children with DSDs had higher scores for anxiety were consistent with the findings from Johansen et al. (17, 18). Also, our findings regarding the increase in both scores of anxiety and depression were in line with results obtained from the adult group (4, 8). For children, our findings indicated a logical link with previous outcomes that children with DSDs had significantly higher total problems reported by their parents (4). However, our findings on depression scores' increase were not consistent with the previous study by Liao et al. (18). And the main findings in the present study contradicted some results from teenagers' self-report scales showing no increase in psychological distress (4, 5, 19). These contradictions suggested the complexity of the mental health status of DSD individuals and might be due to bias that appeared during selection or from the measurements.

Infants born with DSD may undergo long-term medical and surgical management, and some patients with certain forms of DSD and gender designation had life-threatening issues and/or might have increased cancer risk (20–22). Uncertainty about illness and family environment may be important factors related to anxiety and depression in DSD children and their parents. Earlier studies have reported that higher levels of illness uncertainty were related to later decisions that parents made for their child with DSD (23). Independence, entertainment, and emotional expression were less advocated for in Chinese family environments, with excessive emphasis on success (14). Chinese parents attached special importance to their children's success, so success is not only conducive to personal growth but also to family stability (14). Children with DSDs have a significantly different appearance of the external genital device than children without DSDs, so their family members often have severe anxiety. Perez Megan (24) conducted population surveys on mothers (*n* = 76) and fathers (*n* = 63) of DSD children, and evaluated anxiety and depression in children by self-assessment scales, quality of life scales, post-traumatic stress symptom scales, and measurements of appearance satisfaction with the child's genitalia. Lower-income, increased medical expenses, and lack of other children can increase children's psychological distress. The interaction of multiple factors, such as parenting methods, family environment, and school trauma experience, has jointly led to the occurrence, maintenance, and transformation of children's emotional disorders (24, 25).

Children with DSD needed multiple surgeries in the completion of sex distribution and subsequent treatment, which caused great psychological trauma to them. Therefore, long-term psychological evaluation and intervention are needed in these DSD children (26). In addition, children with DSD have an impaired quality of life, but this is not supported by any data (27, 28).

The study's sample size (*n* = 30) appeared relatively small in comparison to prior research. Through MDT-related consultation, the coordination degree is insufficient, increasing the difficulty of enrolled patients. The sample size of this project, therefore, depended on the degree of active participation of these patients. Parents and children gave informed consent through the MDT consultation to complete the assessment content. Domestic newborn screening would detect the vast majority of DSD children in infants, and young children had begun surgical intervention. There were few children who were informed and diagnosed in other age groups. This study was a discussion of anxious mood and its related factors in this particular group. This showed that these special experiences increased the degree of anxiety in children. Awareness of early intervention and related treatment from parents should be raised in the future to lead better outcomes and quality of life for DSD children.

Concerning the age at which a diagnosis is made, the results are conflicting. Some authors suggest that age was not a linear correlation factor in the evolution of psychological distress. Rather, they supported the idea that conditions were silenced or increased by other variables that were more or less actualized depending on life stages (and related to intimate relationships, sexuality, or fertility) (16, 29). In European countries, the age at which the diagnosis was made in real life was correlated to the nature of the etiology itself. Those with late-stage diagnosis and better outcomes were specifically asymptomatic before adolescence and could not be diagnosed earlier (16, 29). With the eugenics policy in China, parents and doctors were already preparing for intervention during their children's fetal life. There were very few children who were informed and diagnosed in childhood.

### Limitations

In this study, patients aged 3–17 years with DSD from one hospital in Hangzhou were recruited. So the Berkson's bias here can hardly be avoided. A relatively high proportion of potential participants who screened positive for emotional disorder on a parental screening form refused to participate. Thus, it is uncertain whether this sample is representative of all children with DSDs in China. The diagnostic assessment of DSD children was quite rigorous, but the evaluation of family environment, parenting mode, depression, and anxiety was assessed by self-report measures. They were susceptible to a variety of biases, for example, recall bias, that was difficult to control in the analysis. In addition, the small sample size and a great majority of women in the participants may weaken the sample representativeness and the power of the study findings to some extent. In addition, the depression and anxiety measures for children (DSRSC, SCARED, and HAMA) assessed children's current symptoms at the time of the survey, which did not reflect their long-term or average level of anxiety and depressive symptoms. Lastly, long-term follow-up studies in the future are needed to clarify the interaction between the mental health status of children with DSD and its associated factors, and to describe the different clinical trajectories of children with DSD.

## Conclusion

This study aims to evaluate the mental status of children with DSD, being valued by parents and professionals. Children with DSD have obvious anxiety problems, which are associated with family environmental factors (entertainment, success, and conflicts) and age. This study expands the profile of children with DSD from the psychological point, and provides the data for their psychological evaluation and intervention for them.

## Abbreviations

DSD, disorders of sexual development; HAMA, Hamilton Anxiety; SCARED, Screen for Child Anxiety Related Emotional Disorders; DSRSC, Depression Self-rating Scale for Children; EMBU, Egna Minnen av Barndoms Uppfostran-own memories of parental rearing practices in childhood; FES-CV, the Home Environment Scale-Chinese version.

## Data availability statement

The original contributions presented in the study are included in the article/supplementary material, further inquiries can be directed to the corresponding authors.

## Ethics statement

The studies involving human participants were reviewed and approved by the Ethics Committee of the Children's Hospital of Zhejiang University School of Medicine. Written informed consent to participate in this study was provided by the participants' legal guardian/next of kin.

## Author contributions

JC and RY designed the protocol and supervised the conduct of the study. JC, WG, and GZ performed the statistical analysis. JC, HT, JY, HG, LS, and DW wrote the first draft of the manuscript. RY, GD, WR, DT, and JF provided valuable advice on the protocol and revised the draft manuscript. All authors approved the final manuscript.

## Conflict of interest

The authors declare that the research was conducted in the absence of any commercial or financial relationships that could be construed as a potential conflict of interest.

## Publisher's note

All claims expressed in this article are solely those of the authors and do not necessarily represent those of their affiliated organizations, or those of the publisher, the editors and the reviewers. Any product that may be evaluated in this article, or claim that may be made by its manufacturer, is not guaranteed or endorsed by the publisher.
